# Impacts of Neoadjuvant Chemotherapy on Perioperative Outcomes in Patients with Bladder Cancer Treated with Radical Cystectomy: A Single High-Volume Center Experience

**DOI:** 10.3390/jpm14020212

**Published:** 2024-02-16

**Authors:** Flavia Proietti, Rocco Simone Flammia, Leslie Claire Licari, Eugenio Bologna, Alfredo Maria Bove, Aldo Brassetti, Gabriele Tuderti, Riccardo Mastroianni, Antonio Tufano, Giuseppe Simone, Costantino Leonardo

**Affiliations:** 1 Department of Urology, IRCCS Regina Elena National Cancer Institute, Via Elio Chianesi 53, 00128 Rome, Italy; 2 Urology Unit, Department of Maternal and Child Health and Urological Sciences, “Sapienza” University of Rome, Viale del Policlinico 155, 00161 Rome, Italy

**Keywords:** neoadjuvant chemotherapy, radical cystectomy, bladder cancer, multimodal approach, complications, morbidity, mortality, robot-assisted surgery

## Abstract

(1) Background: Less than 30% of patients with muscle-invasive bladder cancer (MIBC) receive neoadjuvant chemotherapy (NAC), and reasons for underuse remain unclear. One potential explanation is the concern for the increased risk of perioperative morbidity and mortality. The aim of this study is to investigate the impact of NAC on the risk of detrimental perioperative outcomes in patients with MIBC treated with radical cystectomy (RC). (2) Methods: We identified patients receiving RC for MIBC (T2-4a N0 M0) from 2016 to 2022. Moreover, 1:1 propensity score matching (PSM) was applied between RC alone versus RC plus NAC, and our analysis tested the association between NAC status and peri-operative outcomes. (3) Results: Among the 317 patients treated with RC for identified MIBC, 98 (31%) received NAC. Patients treated with NAC were younger (median yr. 64 vs. 71; *p* < 0.001), with a lower Charlson Comorbidity Index (3 vs. 4; *p* > 0.001), and received more frequently continent urinary diversion (61 vs. 32%, *p* < 0.001). About 43% of patients in each group were treated with robot-assisted radical cystectomy (RARC) with totally intracorporeal urinary diversion (ICUD). After PSM, no differences were detected for the outcomes considered. (4) Conclusions: NAC is not associated with a higher rate of perioperative complications, including patients who received RARC with ICUD.

## 1. Introduction

Muscle-invasive bladder cancer (MIBC) represents a significant clinical challenge accounting for about 20% of newly diagnosed cases of bladder cancer (BCa) [1]. The gold standard treatment for MIBC is radical cystectomy (RC) with bilateral pelvic lymph node dissection. Although open radical cystectomy (ORC) has been the benchmark surgical modality, the landscape of surgical intervention is changing. A notable shift toward robot-assisted procedures, particularly in centers with a high patient volume, is becoming evident [2]. Robot-assisted radical cystectomy (RARC), indeed, has demonstrated its ability to enhance patient safety and achieve oncological outcomes comparable to open surgery. Furthermore, the robot-assisted approach has been associated with notable clinical improvements, including reduced blood loss, fewer perioperative transfusion requirements, and faster post-operative recovery [3].

Regardless of the surgical approach, approximately 50% of patients undergoing RC for MIBC experience the development of metastatic disease within 2 years after their diagnosis with a five-year overall survival (OS) rate for organ-confined MIBC of approximately 50%. Indeed, the presence of muscle invasion represents an unfavorable prognostic indicator. Despite advances in imaging diagnostics [4,5], a portion of patients already harbors micrometastases at the time of diagnosis [6,7].

Several studies, including randomized control trials (RCTs), have provided high-quality evidence (level 1) that the addition of platinum-based combination neoadjuvant chemotherapy (NAC) can improve survival outcomes in comparison to the use of locoregional treatment alone. Current guidelines recommend cisplatin-based NAC in cT2-4 N0 M0 disease, providing a 5–8% advantage in terms of OS at 5 years [8,9]. 

Despite these findings, the use of NAC among eligible patients remains limited, with a treated patient percentage of 15–40% [10,11,12]. Potential reasons have been reported by a survey conducted among members of the Society of Urological Oncology; among these, the most frequent concerns relate to age and comorbidities (54%), potential delay in surgical treatment (35%), and limited benefits (33%). Other factors that may influence the clinical decision to administer NAC include the potential toxicity of chemotherapy (13.6%) and its impact on complications during the peri- and post-operative period (13.6%) [13]. The aim of this study is to investigate the impact of NAC on the risk of detrimental post-operative outcomes in patients with BCa treated with RC for MIBC.

## 2. Materials and Methods

### 2.1. Data Source and Study Population

Following approval from the local ethical committee, patient data from all individuals who underwent RCs for MIBC (cT2-4a N0 M0) in our department from January 2016 to September 2023 were collected from our prospective maintained database and were retrospectively analyzed. This analysis was conducted in accordance with the ethical standards outlined in the 1964 Declaration of Helsinki and its subsequent amendments. Patient data confidentiality was ensured, and the requirement for informed consent was waived. 

Prior to RC, each patient underwent pre-operative transurethral resection of the bladder (TURB), which led to the diagnosis of MIBC. Subsequently, a total body CT scan was performed to exclude metastatic disease or local lymph node spread of the tumor, which are critical considerations for NAC and RC, respectively.

Patients were then referred to an oncologist for cisplatin-based NAC. Patients in the NAC group received either the GC (gemcitabine, cisplatin) or MVAC (methotrexate, vinblastine, adriamycin, cisplatin) regimen for four cycles before surgery [7]. In all patients, the time interval between the end of chemotherapy and surgery did not exceed 6 weeks. All surgical procedures were performed by expert surgeons at a high-volume cancer center. The decision regarding the surgical approach was at the discretion of the operating surgeon. All patients received extended lymph node dissection [7]. Exclusion criteria were (1) variant histology at pathology, (2) suboptimal dose of NAC (<3 cycles or decreased dosage) [14], and (3) history of pelvic irradiation.

### 2.2. Patient Characteristics and Outcomes of Interest

The following baseline characteristics were recorded for each patient: age, gender, age-factored Charlson Comorbidity Index (CCI) [15], American Society of Anesthesiologists (ASA) score [16], body mass index (BMI), pre-operative hemoglobin and estimated glomerular filtration rate (eGFR), administration of antiplatelet/anticoagulant therapy, presence of hydronephrosis, NAC, surgical approach, type of urinary diversion (UD), pathological stage, application of enhanced recovery after surgery (ERAS), and complication rate. Patients were stratified according to NAC status and the following outcomes were analyzed: operation time (OT), length of stay (LOS), 24 h hemoglobin drop, post-operative transfusions, high-grade Clavien–Dindo complications (CD ≥ 3), post-operative acute kidney injury (AKI).

### 2.3. Statistical Analysis

Categorical variables are reported as frequencies and percentages, while continuous variables are reported as medians and interquartile ranges (IQR). Baseline demographics, clinical, and tumor-specific characteristics, as well as perioperative outcomes, were compared using the most appropriate test—Wilcoxon rank-sum test or Pearson’s chi-squared test—across the cohorts.

Propensity score matching (PSM) was performed to reduce the imbalance between groups due to the lack of randomization. With the estimated propensity scores, the ‘RC alone’ group was matched with the ‘RC plus NAC’ group in a 1:1 ratio by using the nearest neighbor matching algorithm without replacement. The covariates considered for each endpoint in the PSM are summarized in the Supplementary File Table S1.

Matching covariate balance was assessed using standardized mean differences. We considered a standardized mean difference of < 0.10 as a negligible imbalance. After PSM, the Wilcoxon test and Pearson’s chi-squared test were performed to assess the association between NAC status and continuous or categorical peri-operative outcomes, respectively.

All analyses were performed using r software (R Foundation for Statistical Computing, Vienna, Austria), version 4.2.2, with the Matchit package. Statistical significance was set at *p* < 0.05.

## 3. Results

### 3.1. Patients’ Characteristics

At our institution, among the 317 patients identified (Table 1), 98 (31%) received NAC prior to RC. The median age of the overall cohort was 69 years (IQR: 62–76), with those in the NAC plus RC group being significantly younger with a median of 64 years (IQR: 59–69) compared to 71 years (IQR: 66–77) in the RC alone group (*p* < 0.001). Patients in the NAC plus RC group also had a lower Charlson Comorbidity Index (3 vs. 4; *p* > 0.001).

Hydronephrosis was present in 24.6% of the overall cohort, with a non-significant difference between groups (*p* = 0.6).

Surgical approaches were evenly distributed between open and robotic methods in both groups, with no significant difference in the choice of approach (*p* > 0.9).

Types of urinary diversion that were different significantly comprise the following: the ureterocutaneous diversion was less common in the NAC plus RC group (19.4%) compared to the RC alone group (42.0%). Conversely, continent urinary diversion was more frequently performed in the NAC plus RC group (61 vs. 32%, *p* < 0.001).

In terms of pathological staging, the NAC group had a higher proportion of organ-confined disease (57.1%) compared to the RC alone group (37.8%) (*p* = 0.001).

About 43% of patients in each group were treated with robot-assisted radical cystectomy (RARC) with totally intracorporeal urinary diversion (ICUD).

### 3.2. Crude Rates of Outcomes of Interest

Before PSM (Table 2), overall and Clavien–Dindo 3–5 complication rates were 60.2 vs. 61.2% and 11.2 vs. 13.2% in NAC plus RC and RC alone groups, respectively (all *p* > 0.05). No statistically significant differences were found between the two groups for the following outcomes: 24 h hemoglobin drop, post-operative transfusions, and post-operative AKI. On the other hand, both operative times and length of hospital stay appear to be longer in patients undergoing NAC (215 vs. 185 min, *p* = 0.005; 7 vs. 6 days, *p* = 0.016).

### 3.3. Outcomes of Interest after Propensity Score Matching

After PSM (Table 3), no differences (all *p* > 0.05) were detected for the outcomes considered according to NAC status (NAC plus RC vs. RC alone): median OT (208 vs. 205 min), median LOS (7 vs. 7 days), 24 h hemoglobin drop (−1.80 vs. −1.80), post-operative transfusions (46 vs. 42.5%), CD 3–5 complications (12.5 vs. 11.2%), and post-operative AKI (11.0 vs. 13.4%).

## 4. Discussion

Randomized controlled trials have demonstrated enhanced overall and disease-free survival rates following the use of NAC [17,18]. Despite this advancement, a considerable number of patients are unable to undergo treatment with cisplatin due to factors such as impaired renal function, suboptimal performance status, or other comorbidities [19].

However, among those who are eligible for treatment with cisplatin, there is a growing trend to administer neoadjuvant chemotherapy for muscle-invasive bladder cancer before radical cystectomy [20,21]. This is evidenced by a retrospective analysis of the International Robotic Cystectomy Consortium (IRCC) database by Aldhaam and colleagues, which revealed a significant increase in the adoption of NAC, from 10% in the period of 2006–2007 to 42% in 2016–2017 [21]. As indicated in Table 4, the percentage of patients receiving NAC was approximately 10% of the total cohort in population-based studies published in 2014 [22,23], and the figure increased to around 40% in more recent series [24].

International guidelines recommend the use of NAC, yet its application remains suboptimal [8]. A contributing factor may be the potential influence on peri- and post-operative morbidity and mortality. Moreover, the timing and quality of radical cystectomy still represent crucial aspects in achieving a cure for patients with MIBC. Hence, systemic treatment and its adverse events should not compromise the timing and extent of surgery, should not increase post-operative morbidity and mortality, and should not influence urinary diversion selection [25].

Our research contributes to the growing body of evidence assessing NAC effects on perioperative outcomes. Evaluating the impact of NAC on the risk of complications is challenging without a randomized controlled trial, given the significant confounding biases that could be present. This challenge stems from the overlap between patient characteristics that dictate eligibility for NAC and those that affect complication risks. We attempted to minimize these biases by performing PSM analysis prior to each analysis of outcomes.

Consistent with the findings from previous retrospective studies [22,23,24,26], our analysis did not find any statically differences in the outcomes of interest (Table 3). For instance, our rate of in-hospital Clavien–Dindo Grade 3–5 complications was comparable, with 12.5% in the cohort receiving NAC plus RC versus 11.2% in the RC alone group. This parity in outcomes is also confirmed by studies examining the 30-day and 90-day post-operative outcomes of these treatments (Table 4) [22,23,24,26]. It is important to underline that our study includes patients who underwent RARC with totally intracorporeal urinary diversion (approximately 43%), and this does not represent a factor capable of influencing the outcome.

Interestingly, in our series, 61% of patients undergoing multimodal treatment receive an ileal neobladder, compared to 31% of those undergoing surgery alone. This pattern aligns with the findings from other studies with a similar design, where patients undergoing NAC are more frequently the chosen candidates for a continent diversion [21,23,24,27,28]. This tendency could be explained by demographic and health status factors. Patients undergoing NAC are, on average, younger by about 4 years in the examined series, and generally have fewer comorbidities, despite potential variability in health indicators such as ASA, CCI, and the modified Frailty Index. In such patients, who are more suitable candidates for continent urinary diversion, specialists may feel more confidential in recommending a multimodal therapeutic approach [21,22,26,27,28,29,30].

According to our findings, NAC status does not affect intraoperative time, corroborating with the results of other studies [22,27]. However, the consistency of these data varies, as some reports have found a statistically significant reduction in the operative time for patients undergoing RC alone [21,24,26]. This significant finding suggests that the observed pathological downstaging should be attributed to the therapeutic efficacy of NAC rather than selection bias, reinforcing the value of NAC in the treatment protocol for muscle-invasive bladder cancer.

From a clinical perspective, our observations indicate that neoadjuvant chemotherapy should be strongly considered by clinicians when it is clinically necessary to improve the overall survival rates of patients with muscle-invasive bladder cancer. However, it is necessary to emphasize that a careful selection of patients is crucial in order to optimize the balance between the benefits and side effects of neoadjuvant chemotherapy [31]. Particular caution should be exercised with older patients and those with pre-existing comorbidities, especially impaired renal function, as these factors not only contraindicate the use of certain chemotherapeutic agents but also heighten the risk of post-operative complications [32].

Despite our comprehensive approach, our study is subject to certain limitations. The retrospective design inherently carries potential for selection bias and data omissions, although we have aimed to mitigate this by prospectively collecting data.

The sample size may also limit this study’s power. Additionally, the predominance of younger patients with fewer comorbidities in the NAC plus RC group could bias outcomes favorably. We addressed this by employing propensity score matching to balance the groups based on their baseline characteristics.

Furthermore, there may be inherent time bias, where patients adversely affected by NAC who could not proceed to RC are not represented in this study’s analysis. Such data were not available within our cohort.

Finally, the findings are derived from a high-volume center, which may not be generalizable to all clinical settings.

**Table 4:** Baseline characteristics and outcomes of patients with MIBC treated with neoadjuvant chemotherapy plus radical cystectomy versus radical cystectomy alone: literature review.

Authors	Johnson et al. [22]	Gandaglia et al. [23]	Salminen et al. [30]
Year	2014	2014	2018
Study design	Retrospective	Retrospective	Retrospective
Population	Population-based	Population-based	Population-based
NAC/Total RC, n/n (%)	78/878 (8.9%)	416/3760 (11.1%)	214/1385 (15%)
	**RC + NAC vs. RC alone**	**RC + NAC vs. RC alone**	**RC + NAC vs. RC alone**
Baseline characteristics			
Age, years	65 vs. 70 (*p* = 0.001)	73 vs. 75	64.5 vs. 68.5
Comorbidity, %	ASA1–2: 33 vs. 26% (*p* = 0.002)	CCI: 39.4 vs. 37.7%	ASA1–2: 44 vs. 37%
Pre-operative renal function	Cr: 1.1 vs. 1.0 (*p* = 0.13)	NA	eGFR: 72 vs. 68
Pre-operative hemoglobin, g/dL	NA	NA	NA
Performed LND, %	NA	NA	NA
Extended LND, %	NA	NA	NA
Continent UD, %	NA	18 vs. 17.2 (*p* = 0.02)	33 vs. 18%
Pathological T0-stage, %	NA	NA	NA
Minimally invasive, %	NA	NA	None
Intracorporeal UD, %	NA	NA	NA
Outcomes			
Operative time, min	363 vs. 345 * (*p* = 0.24)	NA	NA
Length of stay, days	9.3 vs. 11.3 * (*p* = 0.02)	NA	13 vs. 14
Post-operative transfusions, %	38.5 vs. 51.8% (*p* = 0.04)	32.9 vs. 33.8% (*p* = 0.7)	NA
In-hospital complications, %			
Overall	NA	NA	NA
CD 3–5	NA	NA	NA
30-day complications, %			
Overall	55.1 vs. 51.8% (*p* = 0.57)	NA	NA
CD 3–5	NA	NA	NA
90-day complications, %			
Overall	NA	71.9 vs. 72.7 * (*p* = 0.7)	34 vs. 46%
CD 3–5	NA	NA	NA
**Authors**	**Milenkovic et al.** [26]	**Aldhaam et al.** [21]	**Arora et al.** [27]
Year	2019	2019	2022
Study design	Retrospective	Retrospective	Retrospective
Population	Single center	Population-based	Multi center
NAC/Total RC, n/n (%)	102/491 (20.8%)	298/1156 (25.7%)	968/3113 (31.1%)
	**RC + NAC vs. RC alone**	**RC + NAC vs. RC alone**	**RC + NAC vs. RC alone**
Baseline characteristics			
Age, years	62.5 vs. 68.5 (*p* < 0.001)	67 vs. 68 (*p* = 0.01)	66 vs. 70 (*p* < 0.001)
Comorbidity, %	CCI 0: 60.8 vs. 45.5% (*p* = 0.002)	ASA ≥ 3: 62 vs. 55% (*p* = 0.02)	ASA 1–2: 62 vs. 56% (*p* = 0.01)
Pre-operative renal function	Cr: 1.1 vs. 1.2 (*p* = 0.016)	NA	NA
Pre-operative hemoglobin, g/dL	12.6 vs. 13.7 (*p* < 0.001)	NA	NA
Performed LND, %	98.04 vs. 90.49% (*p* < 0.001)	NA	100 vs. 100%
Extended LND, %	60.8 vs. 36.7% (*p* < 0.001)	NA	NA
Continent UD, %	25.9 vs. 35.2% (*p* = 0.61)	20 vs. 15 (*p* = 0.02)	35.3 vs. 25.5% (*p* < 0.001)
Pathological T0-stage, %	27.4 vs. 13.1% (*p* < 0.001)	24 vs. 10% (*p* < 0.01)	NA
Minimally invasive, %	None	All	38.8 vs. 25.3 (*p* < 0.001)
Intracorporeal UD, %	None	70 vs. 53% (*p* < 0.01)	16.2 vs. 11.5% (*p* = 0.06)
Outcomes			
Operative time, min	217.5 vs. 210.0 (*p* = 0.002)	372 vs. 360 (*p* = 0.03)	300 vs. 285 (*p* = 0.08)
Length of stay, days	18 vs. 19 (*p* = 0.08)	8 vs. 8 (*p* = 0.85)	11 vs. 12 (*p* < 0.001)
Post-operative transfusions, %	17.6 vs. 11.5% (*p* = NA)	25 vs. 13% (*p* < 0.01)	NA
In-hospital complications, %			
Overall	NA	NA	NA
CD 3–5	NA	NA	NA
30-day complications, %			
Overall	68.6 vs. 66.0% (*p* = 0.15)	NA	53.2 vs. 54.6% (*p* = 0.4)
CD 3–5	11.7 vs. 11.8% (*p* = 0.98)	NA	15.5 vs. 16.5% (*p* = 0.6)
90-day complications, %			
Overall	NA	43 vs. 37% (*p* = 0.06)	59 vs. 58.5% (*p* = 0.5)
CD 3–5	NA	21 vs. 18% (*p* = 0.23)	20.5 vs. 19.7% (*p* = 0.6)
**Authors**	**Hoeh et al.** [28]	**Riveros et al.** [24]	**Present study**
Year	2022	2022	2023
Study design	Retrospective	Retrospective	Retrospective
Population	Population-based	Population-based	Single center
NAC/Total RC, n/n (%)	805/4347 (19%)	669/1582 (42.2%)	98/317 (31%)
	**RC + NAC vs. RC alone**	**RC + NAC vs. RC alone**	**RC + NAC vs. RC alone**
Baseline characteristics			
Age, years	67 vs. 70 (*p* < 0.001)	67 vs. 72 (*p* < 0.001)	64 vs. 71 (*p* < 0.001)
Comorbidity, %	CCI 0–1: 90 vs. 84% (*p* < 0.001)	ASA ≤ 2: 23.9 vs. 21.0% (*p* = 0.17)	ACCI: 3 vs. 4 (*p* < 0.001)
Pre-operative renal function	NA	Cr (mg/dL) 1.11 vs. 1.05 (*p* = 0.2)	eGFR (mL/min): 76 vs. 68 (*p* = 0.004)
Pre-operative hemoglobin, g/dL	NA	NA	12.7 vs. 13.3 (*p* = 0.006)
Performed LND, %	94 vs. 91% (*p* = 0.005)	98.4 vs. 96.8% (*p* = 0.2)	All
Extended LND, %	NA	NA	All
Continent UD, %	7 vs. 6% (*p* = 0.007)	19.4 vs. 12.9% (*p* = 0.002)	61.2 vs. 34.2% (*p* < 0.001)
Pathological T0-stage, %	NA	21.5 vs. 6.5% (*p* < 0.001)	NA
Minimally invasive, %	40 vs. 40% (*p* = 0.8)	21.1 vs. 17.2% (*p* = 0.07)	42.9 vs. 42.9% (*p* > 0.9)
Intracorporeal UD, %	NA	NA	All
Outcomes			
Operative time, min	NA	343 vs. 303 (*p* < 0.001)	208 vs. 205 * (*p* = 0.5)
Length of stay, days	6 vs. 7 (*p* < 0.001)	NA	7 vs. 7 (*p* = 0.6)
Post-operative transfusions, %	13 vs. 12% (*p* = 0.7)	31.9 vs. 30.8% * (*p* = 0.7)	42.5 vs. 46.0% * (*p* = 0.60.01)
In-hospital complications, %			
Overall	63 vs. 65% (*p* = 0.3)	NA	60.2 vs. 61.2% (*p* = 0.9)
CD 3–5	NA	NA	12.5 vs. 11.2% * (*p* = 0.8)
30-day complications, %			
Overall	NA	NA	NA
CD 3–5	NA	24.5 vs. 20.1% * (*p* = 0.14)	NA
90-day complications, %			
Overall	NA	NA	NA
CD 3–5	NA	NA	NA

* Adjusted value. ACCI = age-adjusted Charlson Comorbidity Index; CCI = Charlson Comorbidity Index; CD = Clavien–Dindo; LND = lymph node dissection; NA = not assessed; NAC = neoadjuvant chemotherapy; RC = radical cystectomy; UD = urinary diversion.

## 5. Conclusions

Despite the limitations of this study, we can conclude that, in selected patients, NAC exposure is not associated with a higher risk of short-term complications and in-hospital mortality nor when a setting of minimally invasive surgery is considered. Therefore, it is reasonable to consider NAC as a safe option for patients with MIBC who undergo ORC or RARC, both with extracorporeal and intracorporeal diversion. Additional efforts are required to improve the adherence to guidelines among healthcare professionals and patients.

## Figures and Tables

**Table 1:** Demographics and clinical characteristics of patients with muscle-invasive bladder cancer treated with radical cystectomy, stratified by neoadjuvant chemotherapy status.

	Overall,	NAC plus RC,	RC alone,	*p*-Value ^1^
	n = 317	n = 98 (31%)	n = 219 (69%)	
Age, yr (median, IQR)	69 (62–76)	64 (59–69)	71 (66–77)	<0.001
Male gender, n (%)	243 (76.7%)	77 (78.6%)	166 (75.8%)	0.6
BMI, kg/m^2^ (median, IQR)	25.7 (24.1–28.1)	26.1 (24.2–27.8)	25.6 (24.0–28.2)	0.6
CCI, n (%)	4 (3–5)	3 (2–4)	4 (3–5)	<0.001
ASA score, n (%)	2 (2–3)	2 (2–3)	2 (2–3)	0.1
Antiplatelet/Anticoagulant therapy, n (%)	130 (41%)	31 (31.6%)	99 (45.2%)	0.023
Presence of hydronephrosis, n (%)	78 (24.6%)	26 (26.5%)	52 (23.7%)	0.6
Pre-operative Hb, g/dL (median, IQR)	13.1 (11.6–14.3)	12.7 (11.5–13.7)	13.3 (11.8–14.4)	0.006
Pre-operative eGFR, mL/min (median, IQR)	71 (54–88)	76 (61–92)	68 (51–86)	0.004
Surgical approach, n (%)				>0.9
Open	181 (57.1%)	56 (57.1%)	125 (57.1%)	
Robotic	136 (42.9%)	42 (42.9%)	94 (42.9%)	
Urinary diversion, n (%)				<0.001
Ureterocutaneous	111 (35.0%)	19 (19.4%)	92 (42.0%)	
Ileal Conduit	71 (22.4%)	19 (19.4%)	52 (23.7%)	
Orthotopic Neobladder	135 (78.2%)	60 (61.2%)	75 (34.2%)	
Pathological stage, n (%)				0.001
Organ confined *	139 (43.8%)	56 (57.1%)	83 (37.8%)	
Non-organ confined	178 (56.2%)	42 (42.9%)	136 (62.1%)	
ERAS application, n (%)	248 (78%)	73 (74.5%)	175 (79.9%)	0.3

^1^ Wilcoxon rank-sum test; Pearson’s chi-squared test. NAC = neoadjuvant chemotherapy; RC = radical cystectomy; BMI = body mass index; CCI = Charlson Comorbidity Index, ASA = American Society of Anesthesiologists; Hb = hemoglobin; eGFR = estimated glomerular filtration rate; ERAS = enhanced recovery after surgery. * Organ confined (pT ≤ 2 and pN0); non-organ confined (pT ≥ 3 and/or pN ≥ 1).

**Table 2:** Perioperative outcomes of patients with muscle-invasive bladder cancer treated with radical cystectomy, stratified by neoadjuvant chemotherapy status.

	Overall, n = 317	NAC plus RC, n = 98 (31%)	RC alone, n = 219 (69%)	*p*-Value ^1^
Operative time, min (median, IQR)	205 (145–270)	215 (162–285)	195 (130–261)	0.005
Length of Stay, days (median, IQR)	7 (5–9)	7 (6–10)	6 (5–9)	0.016
Any complications, n (%)	193 (60.9%)	59 (60.2%)	134 (61.2%)	0.9
CD 1–2 complications, n (%)	184 (58.0%)	55 (56.1%)	129 (58.9%)	0.6
CD 3–5 complications, n (%)	40 (12.6%)	11 (11.2%)	29 (13.2%)	0.3
24 h Hb drop, g/dL (median, IQR)	−2.00 (1.30–3.00)	−1.75 (1.10–2.75)	−2.10 (1.30–3.00)	0.067
Post-operative transfusions, n (%)	133 (42.0%)	41 (41.8%)	92 (42.0%)	>0.9
Post-operative AKI, n (%)	38 (12.0%)	13 (13.3%)	25 (11.4%)	0.6

^1^ Wilcoxon rank-sum test; Pearson’s chi-squared test. NAC = neoadjuvant chemotherapy; RC = radical cystectomy; Hb = hemoglobin; CD = Clavien–Dindo; AKI = acute kidney injury.

**Table 3:** Outcomes of interest after propensity score matching of patients with muscle-invasive bladder cancer treated with radical cystectomy, according with NAC status.

	NAC plus RC	RC alone	*p*-Value ^1^
Operative time, min (median, IQR)	N = 82 208 (160–284)	N = 82 205 (145–284)	0.5
Length of Stay, days (median, IQR)	N = 79 7 (6–10)	N = 79 7 (5–9)	0.6
CD 3–5 complications, n (%)	N = 80 10 (12.5%)	N = 80 9 (11.2%)	0.8
24 h Hb drop, g/dL (median, IQR)	N = 87 −1.80 (1.10–2.85)	N = 87 −1.80 (1.25–2.85)	0.6
Post-operative transfusions, n (%)	N = 87 41 (41.8%)	N = 87 92 (42.0%)	0.6
Post-operative AKI, n (%)	N = 82 9 (11.0%)	N = 82 11 (13.4%)	0.6

^1^ Wilcoxon rank-sum test; Pearson’s chi-squared test. NAC = neoadjuvant chemotherapy; RC = radical cystectomy; Hb = hemoglobin; CD = Clavien–Dindo; AKI = acute kidney injury.

## Data Availability

The data presented in this study are available upon request from the corresponding author. The data are not publicly available due to restrictions.

## Supplementary Materials

The following supporting information can be downloaded at: https://www.mdpi.com/article/10.3390/jpm14020212/s1, Table S1: Considered covariates for each variable in propensity score matching.
