# Supplementary material for: Impacts of Neoadjuvant Chemotherapy on Perioperative Outcomes in Patients with Bladder Cancer Treated with Radical Cystectomy: A Single High-Volume Center Experience
Source: J Pers Med. 2024 Feb 16;14(2):212. doi: 10.3390/jpm14020212 (PMC10890459; doi:10.3390/jpm14020212)
Supplement: Supplementary file 1 [file jpm-14-00212-s001.zip › jpm-2858668-supplementary.pdf]

Table S1. Covariates considered for each variable in propensity score matching

Variable	Covariates
**CD 3-5**	CCI, BMI, Stage, UD, Approach
**OT**	CCI, BMI, Stage, UD, Approach, Presence of hydronephrosis
**LOS**	CCI, BMI, Stage, UD, Approach, ERAS
**24-hour drop hemoglobin**	CCI, BMI, Stage, Transfusions, preoperative HB, Antiplatelets/Anticoagulant therapy
**Transfusions**	CCI, BMI, Stage, preoperative HB, Antiplatelets/Anticoagulant therapy
**AKI**	CCI, BMI, preoperative eGFR, Presence of hydronephrosis, UD, Approach

CD = Clavien Dindo; CCI = Charlson Comorbidity Index; BMI = Body Mass Index; UD = Urinary Diversion; OT = Operation Time; LOS = Length of Stay; ERAS = Enhanced Recovery After Surgery; HB = Hemoglobin; AKI = Acute Kidney Injury; eGFR = estimated Glomerular Filtration Rate
